# Effectiveness and Tolerability of a Two-Week Hypocaloric Protein-Rich Diet Prior to Obesity Surgery with Two Different Diet Interventions: a Prospective Randomized Trial

**DOI:** 10.1007/s11695-022-06180-z

**Published:** 2022-07-18

**Authors:** Undine Gabriele Lange, Yusef Moulla, Tatjana Schütz, Matthias Blüher, Veronika Peter, Edward Shang, Arne Dietrich

**Affiliations:** 1 Clinic for Visceral, Transplant, Thoracic and Vascular Surgery, Leipzig University, Liebigstr. 20, 04103 Leipzig, Germany; 2 Integrated Research and Treatment Center (IFB) Adiposity Diseases, 04103 Leipzig, Germany; 3 Clinic for Internal Medicine, Leipzig University, 04103 Leipzig, Germany; 4 Clinic for Nephrology, Rheumatology and Diabetology, Halle University, 06108 Halle, Germany

**Keywords:** Preoperative very low-calorie diet, Liver volume reduction, Compatibility of preoperative diets

## Abstract

**Purpose:**

Preoperative very low-calorie diets (VLCDs) have been shown to reduce liver volume and improve bariatric surgery safety. Here, we compare two VLCD that differ in macronutrient composition.

**Material/Methods:**

Ninety patients awaiting obesity surgery were included in a prospective, open-label, randomized mono-centre trial comparing the effects of 2-week preoperative VLCDs: BCM Diät™ (diet 1) versus Optifast™ (diet 2).

**Results:**

Data from 33 patients in diet 1 and 36 in diet 2 could be analysed. There was no significant difference between the two diet intervention arms on outcome parameters. Overall, both VLCD strategies led to a mean weight reduction of 5.24 [4.72–5.76] kg (*p* < 0.001), mean excess weight loss was 8.2 [7.4–9.1] % (*p* < 0.001). BMI reduction was 1.81 [1.63–1.99] kg/m^2^ (*p* < 0.001). Over all patients, the liver volume was reduced by 397 [329–466] ml (*p* < 0.001), which corresponds to 14.6 [12.4; 16.8] %. Liver fat content was significantly reduced by 18.35 [8.98–27.71] %. Reduction of body weight correlates with liver volume loss.

In addition, hip/waist circumferences, body fat and fat-free mass decreased significantly. We found an increase of ALAT/ASAT and a significant decrease of triglycerides, LDL-cholesterol and HbA1c. Parameters of inflammatory were significantly reduced upon VLCD.

**Conclusion:**

Independently of the macronutrient composition, VLCD leads to a significant decrease of body weight, reduction of liver volume and improved parameters of inflammation, glucose and lipid metabolism. Preoperative diets are widely used in conditioning; however, VLCD should be considered as option for patients with obesity undergoing other abdominal surgeries.

**Graphical abstract:**

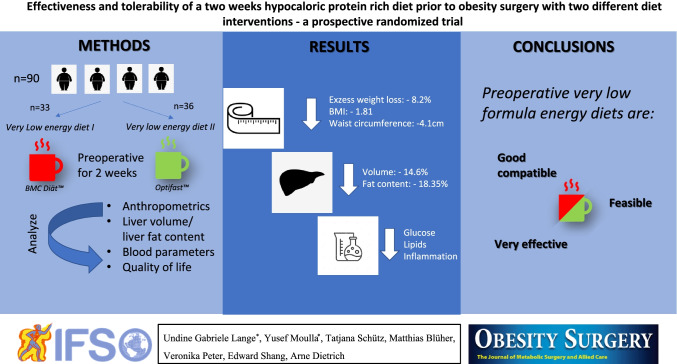

## Introduction/Purpose

Overweight (equal to a body mass index (BMI) ≥ 25 kg/m^2^) and obesity (BMI ≥ 30 kg/m^2^) belong to the greatest public health challenges of the twenty-first century. According to the World Health Organization (WHO) data from 2016, a total of 26% of German adults and 10% of adolescents (10–19 years) have obesity [1]. Until 2025, obesity prevalence in Germany may rise up to 30% in adults, since 67.1% of men and 53% of women are overweight [2].

Overweight and obesity affect the body in many different ways: the development of diabetes, cardiovascular diseases, osteoarthrosis and fatty liver disease are the most common obesity-related disorders. The condition of fatty liver degeneration is summarized in terms of “non-alcoholic fatty liver disease” (NAFLD). The most severe form of this chronic liver disease is non-alcoholic steatohepatitis (NASH) which can progress into fibrosis, cirrhosis, liver failure or liver cancer. These serious and live-shortening comorbidities are increasing in the population due to the pandemic situation of obesity and type 2 diabetes; both are the most relevant NAFLD causes.

The high prevalence of NAFLD [3, 4] in patients with obesity is associated with more complicate abdominal surgeries due to impaired visibility of the gastroesophageal junction, liver vulnerability with risk of bleeding and several others [5]. Therefore, strategies to reduce liver volume prior to abdominal surgery are part of pre-conditioning to minimize morbidity and improve outcome [6]. In this context, preoperative, very low-calorie diets were found to be effective and feasible [6]. Therefore, an energy-restricted diet prior to bariatric surgery is recommended by the American and German societies for obesity (Guideline recommendation Grade B, best evidence level 1) [7, 8]. However, the impact of macronutrient composition and the type of diet [9–11] on perioperative outcomes [12] or long-term effects [13] are discussed controversially.

Most commonly, either a 2-week low-energy diet (LED) or very low-calorie diets (VLCDs) with a total energy intake of < 1000 kcal/d are used, because these interventions were shown to reduce visceral fat and liver volume. Consequently, a better surgical access, shorter operating times, less blood loss and therefore a safer operation are conceivably and partly confirmed [14–17]. For example, no shortening of the operating time was observed by van Nieuwenhove et al., but the overview during the operation was classified as better [15]. Among other things, liver shrinking has been found to decrease collagen production in wounds [18]. However, the extent to which this has an effect on reducing wound infections remains to be investigated. Moreover, a preoperative diet is a test of compliance, and by taking proofed products, pre-existing deficiencies (protein, micronutrients) might be corrected. It has been shown that VLCDs are more effective in terms of required time and weight loss compared to low-calorie diets [19]. Further limiting energy intake does not lead to better effects on reducing liver volume [20]. Although the effects of preoperative low-energy diets before bariatric surgery have been thoroughly studied, a widely accepted standard protocol does not exist so far.

In our study, we compare two commercially available liquid low-energy formula diets in a randomized, prospective setting. Our primary endpoint was reduction of liver volume and liver fat content measured by magnetic resonance imaging (MRI). Secondary endpoints included overall weight reduction, excess weight loss, waist circumference reduction, visceral and subcutaneous adipose tissue mass, laboratory parameters, adherence to and acceptance of the diets and side effects.

## Materials and Methods

### Study Setting

The study was prospective, open-label, two-armed, randomized controlled mono-centre trial in an obesity treatment centre in a university hospital setting. Patients scheduled for bariatric surgery were consecutively screened and included into the study when they met the inclusion/exclusion criteria.

### Inclusion and Exclusion Criteria

Study inclusion criteria were as follows: women and men, age 18–65 years; BMI ≥ 40 kg/m^2^ or BMI ≥ 35 kg/m^2^ and at least one obesity-related comorbidity; indication for bariatric surgery and written informed consent. Exclusion criteria were as follows: any chronic inflammatory or malignant disease, drug abuse, alcoholism (intake > 20 g/day), psychiatric disorders, untreated thyroid dysfunction, pregnant or nursing women, systemic corticosteroid therapy (prednisolone > 2 mg/day), lactose intolerance, concomitant participation in any other interventional trial, contraindication to MRI or MRI not feasible (e.g. person was too large for the radiological equipment) and concomitant therapy with orlistat, psychiatric medication or chemotherapies.

Forty-five patients were included into diet 1-arm, but 12 patients were lost to follow-up due to MRI-associated problems (*n* = 5), study termination at patient’s request (*n* = 3), diet-associated problems (*n* = 2), not reachable (*n* = 1) or obesity surgery was not performed (*n* = 1). Forty-five patients were randomized into diet 2-arm, but 9 patients were lost to follow-up (MRI-associated problems (*n* = 4), study termination at patient’s request (*n* = 4), not reachable (*n* = 1)).

### Diet Intervention

#### Experimental Intervention

Low-energy diet 1 (diet 1, BCM Diät™, PreCon GmbH, Darmstadt) containing 75-g protein, 28-g fat and 80-g carbohydrate, energy intake ≙ 913 kcal/d.

#### Control Intervention

Low-energy diet 2 (diet 2, Optifast™, Nestle HealthCare Nutrition GmbH, München) containing 58-g protein, 19-g fat and 111-g carbohydrate, energy intake ≙ 841 kcal/d.

At BCM Diät™ (diet 1) soups with flavours (vegetables, potato leek, broccoli, chicken curry and tomato), shakes with flavours (chocolate, cappuccino, strawberry, banana and vanilla) and bars with flavours (chocolate, chocolate crisp and cranberry crisp) are offered. Patients should consume daily one soup, two shakes and one bar. Soups and shakes are prepared with 100 ml skimmed fresh, alternatively with 400 ml buttermilk, 300 ml sour milk, Kefir or yogurt or 200 mg low-fat curd. The used milk product and its fat content was noted and included in the calculation.

In the context of Optifast II™ diet (diet 2), patients are supposed to consume four shakes (flavours: chocolate, coffee, vanilla, strawberry) and one soup (flavours: potato leek or tomato) daily. Shakes are prepared with 200 ml of cold water and soups with 250 ml water (temperature until 60 °C).

In addition to the diet products, an intake of 200-g low-starch vegetables per day was allowed in both groups. Patients get for this an overview over the recommended vegetables with calorie information. The patients have been instructed to drink 2 l calorie-free beverages daily. All study participants completed a food diary.

### Sample Size

Published results suggested that liver volume reductions of about 200 ml with standard deviations of 400 ml could be expected [21, 22]. The sample size was based on detecting an effect size of 0.8. Using these criteria for the main outcome parameter, 68 patients would have been required to achieve estimated statistical power of 80%, a conservative estimate compared to the power attained by the ANCOVA used. Based on the clinical setting and the attrition in Lewis et al. [23], we assumed a low drop-out rate of about 5% and planned to enrol 72 patients. This number was increased to 90 in an amendment, since a fairly large proportion of patients did not fit into the MRI machine and could not provide data for the primary endpoint.

### Recruitment and Randomization

Participants were recruited from March 2015 to July 2017 from the list of candidates for laparoscopic Roux-en-Y gastric bypass (LRYGB) and laparoscopic sleeve gastrectomy (LSG) after undergoing multidisciplinary supervision carried out by endocrinologists, surgeons, psychologists, anaesthetists and dietitians.

After explaining the project, volunteers were randomly assigned to one of the two dietary interventions. The randomization was performed computer-assisted, using Pocock’s minimization algorithm. A randomization ratio of 1:1 was used. The randomization was stratified according to gender, age (< 45, >  = 45 years) and initial BMI (< 40, >  = 40 kg/m^2^).

### Anthropometric Parameters

Gender, age, BMI, waist, hip, upper arm and neck circumferences, blood pressure, diet protocols and concomitant medication were taken.

### MRI Examinations

Liver volume will be assessed by a series of 10-mm gradient echo T1-weighted (TE 4.6 ms, TR 550 ms) trans-axial abdominal scans encompassing the entire liver acquired in a single breath-hold using a 1.5 T magnet (Philips Intera Master, Best, Netherlands). Using Philips EasyVision software, the liver margins in each image are manually outlined and the total volume of the liver calculated by multiplying the measured surface area of each slice by the slice thickness and summing the volume of each slice. This type of liver volume measurement has similarly already been carried out by other study groups and is based on the protocol established by Schiano et al. [24].

Liver fat content was assessed by 1H-NMR spectroscopy (blinded analyses). A 20 × 20 × 20-mm voxel was identified within the liver parenchyma using a single axial abdominal image. Measurements were performed in the right (segments IV and VII) and left (segment III) lobe of the liver. H-NMR spectra were acquired using a point resolution spectroscopy sequence with (TE 31 ms, TR 3000 ms, 80 measurements) and without (TE 31 ms, TR 3000 ms, 32 measurements) water suppression. The quantity of intrahepatocellular lipid (IHCL) in the liver voxel is represented by the area under the relevant peak in a Fourier-transformed free induction decay. This area is calculated by applying Lorentzian curve fitting and by the AMARES non-linear least squares algorithm. The results are expressed as a ratio of the area under the IHCL peak in the water suppressed sequence to that of the sum of the areas under the IHCL and water peaks derived from similar analysis of data from the unsuppressed sequence.

The visceral adipose tissue and subcutaneous fat areas are determined by MRI scans. The MRI scan will consist of 10 slices between L4 and L5. Assessment of the fat area was performed by a radiologist blinded to the randomized intervention. The mean fat area of the 10 slices was computed and further used for analysis as described previously.

### Bioelectrical Impedance Analysis (BIA)

Percentage body fat, total body water (TBW) and body cell mass (BCM) were measured by bioelectrical impedance analysis (BIA).

BIA is non-invasive, relatively inexpensive, does not expose to ionizing radiation and has very limited intra-observer variations. BIA works well in healthy subjects and in patients with chronic diseases with a validated BIA equation that is appropriate with regard to age, sex and race. In patients with BMI > 40 kg/m^2^, BIA is not well validated but may be used in follow-up measurements. The measurements were performed according to guidelines under standardized conditions using a BIACORPUS RX 4000 analyser (MEDI CAL HealthCare GmbH, Karlsruhe, Germany). Patients are in a supine position, the arms relaxed at the sides but not touching the body and thighs separated. Four pairs of current-inducing and voltage-sensing electrodes (BIAPhaser, MEDI CAL HealthCare GmbH, Karlsruhe, Germany) are attached to the dorsum of both hands and feet, and resistance (R) and reactance (Xc) are measured applying an alternating electric current of 800 mA at 50 kHz. Raw data were analysed using the software BodyComp V 8.4 Professional.

### Blood Samples

Plasma and serum samples were collected in the morning after overnight fasting at the beginning and at the end of the diet.

Samples were investigated for concentrations of glucose, insulin, serum bilirubin, gamma GT (γGT), alkaline phosphatase (AP), ALAT, ASAT, LDL-cholesterol, HDL-cholesterol and triglycerides.

### Liver Biopsy

A liver sample was obtained during surgery in all study participants from the anterior margin left lobe segment 3 that was given to pathology preserved in 10% formalin.

Paraffin embedded tissue sections were stained using haematoxylin–eosin and Masson trichrome protocols. A pathologist blinded to the study analysed the liver biopsy prospectively. The samples were scored regarding steatosis, inflammation, fibrosis and ballooning of hepatocytes, and the NAFLD activity score according to Kleiner et al. was used [25]. Steatosis hepatitis was diagnosed if the patient had > 5% fat in their liver; NASH was confirmed when ballooning of hepatocytes was present.

### Statistical Analysis

#### Primary Analysis

The primary endpoint was evaluated using analysis of covariance (ANCOVA), with the liver volume at 2 weeks as dependent variable, liver volume at baseline as covariate and the randomization group as factor. Sensitivity analyses included the stratification variables as covariates.

#### Secondary Endpoints

Quantitative secondary endpoints (liver fat content, visceral adipose tissue, subcutaneous fat area, body fat mass, laboratory values) were analysed along the same lines as the primary endpoint. Comparisons of continuous variables for highly related clinical endpoints (such as liver fat content from MRT and pathological assessment) were analysed with Bland–Altman techniques.

## Results

A total of 182 patients planned for obesity surgery were screened, and according to the inclusion and exclusion criteria, 90 patients were randomized, 45 into each study arm. At baseline, there were no differences between the two arms, except for the liver fat content (Table 1). Twelve patients in diet 1-arm and 9 in diet 2-arm dropped out from the study. The compliance was high, and the total energy intake was in diet 1 group 895.7 ± 146.2 kcal/day and 867.3 ± 112.5 kcal/day in diet 2 group. Total energy intake was comparable between the two VLCDs (diet 1: 913 kcal/day; diet 2: 841 kcal/day).

**Table 1:** Baseline values at first cursory analysis

	Diet 1 (n = 33)	Diet 2 (n = 36)	p-value
Number of females	22 (64.7%)	25 (69.4%)	0.7
Age (years)	47.2 (11.5)	46.3 (8.8)	0.7
BMI (kg/m^2^)	47.5 (5.5)	47.5 (5.0)	1.0
Waist circumference (cm)	134.2 (14.0)	136.0 (14.3)	0.6
Hip circumference (cm)	143.5 (9.6)	144.5 (13.9)	0.7
Neck circumference (cm)	44.6 (4.8)	44.6 (5.2)	1.0
ASAT (*µ*kat/l)	0.6 (0.3)	0.6 (0.2)	0.4
ALAT (*µ*kat/l)	0.8 (0.4)	0.6 (0.3)	0.1
AP (*µ*kat/l)	1.3 (0.3)	1.4 (0.4)	0.4
*γ*-GT (*µ*kat/l)	0.8 (0.6)	0.8 (0.6)	0.9
Cholesterol (mmol/l)	5.2 (0.8)	5.1 (1.2)	0.8
HDL (mmol/l)	1.3 (0.4)	1.3 (0.3)	0.5
LDL (*µ*mol/l)	3.1 (0.7)	3.1 (1.0)	0.8
Triglycerides (mmol/l)*	2.1 (1.6)	1.9 (1.1)	0.7
Fasting glucose (mmol/l)**	6.2 (1.8)	6.1 (2.2)	1.0
Number with diabetes	16 (48.5%)	17 (47.2%)	0.9
Liver volume (ml)	2687 (691)	2634 (576)	0.7
Liver fat (%)	23 (13)	16 (11)	0.02
Visceral fat (ml)	6573 (3043)	6246 (2807)	0.6

Data are means (standard deviation) or numbers (%)

^*^Four values are missing from the blood fat analysis in the VLCD2

^**^Fasting glucose in unavailable for 3 patients from VLCD1 and 4 from VLCD2

### Anthropometrics

Baseline anthropometric data are shown in Table 2. The 2-week diet led to an overall weight reduction of 5.24 [4.72–5.76] kg (*p* < 0.001). The excess weight loss was 8.2 [7.4; 9.1] %, and the BMI reduction was 1.81 [1.63; 1.99] kg/m^2^ (*p* < 0.001). All body circumferences dropped significantly upon the VLCD intervention. The mean waist circumference reduction was 4.1 [3.3; 4.9] cm (*p* < 0.001), hip 3.3 [2.4; 4.3] cm (*p* < 0.001), neck 1.1 [0.7; 1.4] cm (*p* < 0.001) and upper arm 0.8 [0.3; 1.3] cm (*p* < 0.001). There was no significant difference in any outcome parameter between diet 1 and diet 2.

**Table 2:** Comparison of formula diet 1 and diet 2

	Diet 1	Diet 2
Product	BCM Diät™ (PreCon GmbH, Darmstadt)	Optifast II™ (Nestle HealthCare Nutrition GmbH, München)
	2 shakes, 1 soup, 1 bar per day	4 shakes, 1 soup per day
Protein	75 g	58 g
Fat	28 g	19 g
Carbohydrates	80 g	111 g
Energy content	913 kcal/d	841 kcal/d
	+ 200 g/d low-starch vegetables (see supplement 1) + 2 l/d calorie-free beverages	

### MRI Liver Volume

At baseline, there were no differences between the groups. The total liver volume was 2687.2 ± 691.4 cm^3^ in patients receiving diet 1 and 2634.1 ± 575.8 cm^3^ in patients receiving diet 2 (*p* = 0.73). The left liver lobe had a mean volume of 473.4 ± 234.2 cm^3^.

**Fig. 1:**
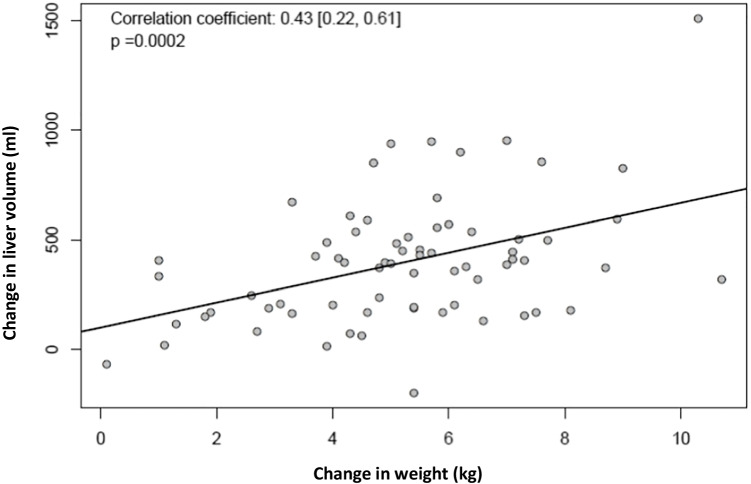
Correlation of liver volume with total weight loss

Over all patients, the liver volume was reduced by 397 [329; 466] ml (*p* < 0.001) or 14.6 [12.4; 16.8] %. After diet 1, liver volume reduction was 457 [349; 565] ml and in group 2 342 [255; 429] (*p* between groups = 0.08). There was a correlation between the reduction of body weight and liver volume (Fig. 1).

### MRI Liver Fat Content

At baseline, the liver fat content was 19.36 ± 12.54%. After both diets, liver fat content dropped significantly 18.35 [8.98; 27.71] % (diet 1: 14.77 [− 2.6; 32.15] %; diet 2: 21.51 [11.64; 31.37] %; *p* between groups = 0.413). For these analyses, only patients with an initial liver fat content of ≥ 5% were included.

**Fig. 2:**
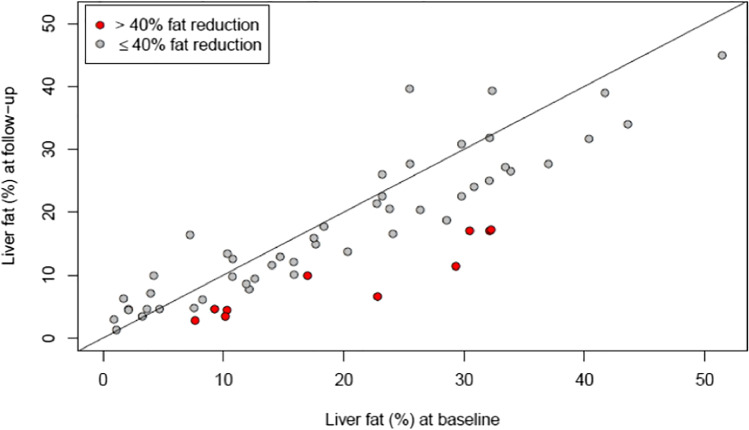
Reduction of the liver fat content during the 2-week diet. Correlation coefficient (*r* =  − 0.34 [− 0.54; − 0.10]; *p* = 0.006)

The diet effects on reducing liver volume correlate with baseline liver fat content (*r* =  − 0.34 [− 0.54; − 0.10], *p* = 0.006; Fig. 2).

### MRI Visceral and Subcutaneous Adipose Tissue Mass

At baseline, mean visceral adipose tissue volume was 6402. ± 2905cm^3^ (diet 1: 6572 ± 3042 cm^3^; diet 2: 6245 ± 2807 cm^3^). Abdominal subcutaneous adipose tissue volume could not be properly assessed, because 94.4% exhibited MRI artefacts.

After VLCDs, visceral adipose tissue volume was reduced by 202 [125; 278] ml (3%) (*p* < 0.001). There was no difference between the diets.

### Bioelectrical Impedance Analysis (BIA)

 BIA analyses revealed a mean decline of body fat of 2.78 ± 1.43 kg (*p* < 0.001), which corresponds to a reduction of 5.7%. There was no significant difference between the two diet groups (*p* = 0.12). Fat-free mass decreased significantly by 2.25 ± 3.78 kg (*p* < 0.001) equivalent to 3.1% without diet group differences (*p* = 0.32).

**Fig. 3:**
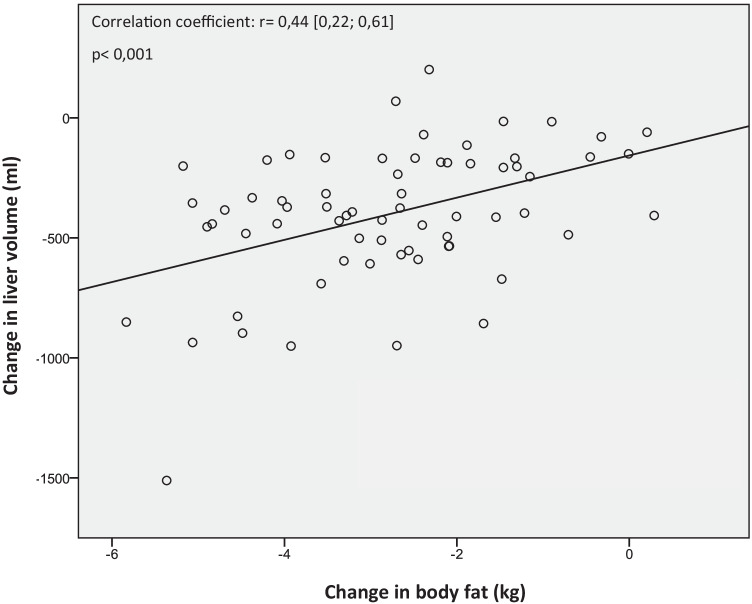
Correlation between change of body fat mass (kg) determined by BIA and change of liver volume (ml) mesured by MRI

There is a moderate correlation between changes of body fat determined by BIA and liver fat content measured by MRI (Fig. 3). Decrease of body fat correlates with reduction of liver volume (*r* = 0.44 [0.22; 0.61], *p* < 0.001) (Fig. 2).

### Serum Parameters

After 2 weeks of VLCD, there was an (median) increase of both ALAT (0.15 (0.35) µkat/l, *p* < 0.001) and ASAT (0.05 (0.19) µkat/l, *p* = 0.003). The number of patients with an increased (pathologic) ALAT value increased from 37.7% at baseline to 62.5% after the diet (ASAT: 24.6 to 35.9%). The De Ritis quotient (ASAT/ALAT) was reduced in both groups (*p* < 0.001) and was below 1 at both time points. AP decreased slightly, but γGT significantly. Total bilirubin increased by 19.6% (*p* = 0.04), in diet 1 0.50 (3.35) µmol/l and in diet 2 um 1.0 (3.5) µmol/l (*p* = 0.19). The number of patients with an increased pathologic bilirubin value dropped from 6 to 4.

Total, LDL and HDL cholesterol and triglycerides dropped significantly (all *p* < 0.001 without differences between diet 1 and diet 2). The liver fat loss correlate with the decrease of total cholesterol (*r* = 0.38 [0.08; 0.0]; *p* = 0.013) and LDL cholesterol (*r* = 0.37 [0.08; 0.59]; *p* = 0.015).

Regarding glucose metabolism: HbA1c dropped from 5.51 (1.38) to 5.35% (1.08), *p* < 0.001; fasting glucose dropped (*p* = 0.028), while fasting insulin values did not change upon the dietary interventions.

Inflammatory parameters dropped: C-reactive protein (CRP) from 8.37 (9.66) to 6.10 (8.33) mg/l (*p* = 0.026). Consequently, the number of patients with increased pathologic CrP dropped from 46 to 37. Leucocyte counts dropped by 0.95 (1.85)10^9^/l (*p* = 0.004).

There was no change in haemoglobin, haematocrit, thrombocytes and erythrocytes.

### Liver Biopsy Histology

**Fig. 4:**
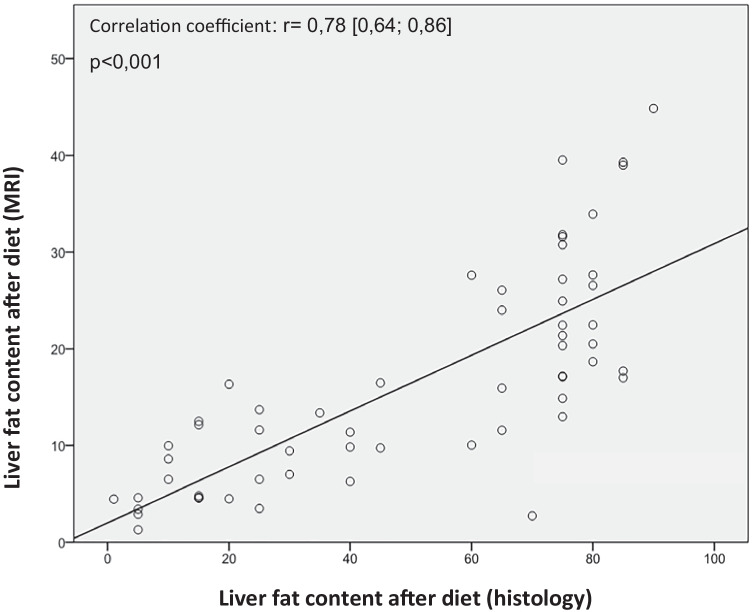
Comparison of liver fat content measured by MRI or histopathological

Liver biopsies could be obtained from 65 patients (94.2%). Almost all patients had fatty liver disease. As shown in Fig. 4, liver fat content measured by MRI correlates with histopathological findings (*r* = 0.78 [0.64; 0.86], *p* < 0.001).

NAFLD was seen in 98.5% of the biopsies, 53.8% showed signs of NASH and fibrosis in 95.4%. NAFLD activity score and fibrosis grade are shown in Table 3.

**Table 3:** NAFLD activity score (grading) and fibrosis grad (staging) after 2 weeks’ diet at the time of operation (total and per cent)

	**Total**	**Diet 1**	**Diet 2**
NAFLD activity score	(*n* = 64)	(*n* = 30)	(*n* = 34)
1	5 (7.8%)	1 (3.3%)	4 (11.8%)
2	11 (17.2%)	6 (20.0%)	5 (14.7%)
3	9 (14.1%)	3 (10.0%)	6 (17.6%)
4	4 (6.8%)	1 (3.3%)	3 (8.8%)
5	11 (17.2%)	3 (10.0%)	8 (23.5%)
6	15 (23.4%)	10 (33.3%)	5 (14.7%)
7	8 (12.5%)	5 (16.6%)	3 (8.8%)
8	1 (1.6%)	1 (3.3%)	0 (0%)
Fibrosis grad	(*n* = 64)	(*n* = 29*)	(*n* = 35)
0	2 (3.1%)	1 (3.4%)	1 (2.9%)
1a	3 (4.7%)	1 (3.4%)	2 (5.7%)
1b	1 (1.6%)	0 (0%)	1 (2.9%)
1c	54 (84.4%)	25 (86.2%)	29 (82.9%)
2	0 (0%)	0 (0%)	0 (0%)
3	3 (4.7%)	2 (6.9%)	1 (2.9%)
4	1 (1.6%)	0 (0%)	1 (2.9%)

^*^In diet 1, one patient was excluded, since fibrosis was not graded

A liver cirrhosis (what was unknown before) was diagnosed in 2 (2.9%) study participants.

### Operation Time and Side Effects

The mean operation time was 159.2 ± 50.7 min (diet 1 165.5 ± 47.1 min; diet 2 153.8 ± 53.7 min (*p* = 0.35), including additional cholecystectomies in some cases). There was no correlation between operation time and liver volume (loss), weight loss, BMI reduction, visceral adipose tissue loss or circumferences drop.

Side effects of the diet were rare (Table 4). There were no serious adverse events during the course of the study.

**Table 4:** Frequency of unexpected adverse events (total and %) during the 2 weeks’ diet

	Total	Diet 1	Diet 2
Obstipation	12 (17.4%)	5 (15.2%)	7 (19.4%)
Diarrhoea	5 (7.2%)	2 (6.1%)	3 (8.3%)
Abdominal pain	5 (7.2%)	2 (6.1%)	3 (8.3%)
Dizziness	5 (7.2%)	3 (9.1%)	2 (5.6%)
Headache	4 (5.8%)	2 (6.1%)	2 (5.6%)
Nausea	4 (5.8%)	1 (3.0%)	3 (8.3%)
Vomiting	2 (2.8%)	1 (3.0%)	1 (2.8%)
Fatigue	2 (2.8%)	2 (6.1%)	0
Flatulence	1 (1.4%)	0	1 (2.8%)
Heartburn	1 (1.4%)	1 (3.0%)	0
Hypoglycaemia	1 (1.4%)	1 (3.0%)	0
Others	4 (5.8%)	2 (6.1%)	2 (5.6%)

## Discussion

The most obvious effect of VLCD for patients is weight loss. The 2-week VLCD led to a significant weight reduction of 5.24 kg, an excess weight loss of 8.2% and a BMI reduction of 1.81 kg/m^2^. Over all patients, all measured body circumferences dropped significantly. The mean waist circumference reduction was 4.1 cm. Bioimpedance analysis showed in both groups a significant loss of body fat of 5.7%, and the fat-free mass was also significantly reduced by 3.1%; those results were comparable to other studies [26, 27]. There was no significant difference between the two groups.

A modest weight loss of 5% from baseline is generally accepted as a “clinically meaningful” amount [28] and has proven beneficial effects on diabetes, cardiovascular disease risk factors (e.g. hypertension), osteoarthritis and NASH [29]. Especially the hepatic and metabolic effects and the compatibility were subjects of our comparative study of two VLCD diets before bariatric surgery.

One effect of preoperative diets is liver reduction by 5 to 20% of the initial volume [30]. In accordance with previous data, we could achieve a liver volume reduction of 14.6% with a very low-calorie diet 2 weeks prior to surgery. In response to diet 1, a trend for a greater loss of − 115 ml liver volume was detected. Clinically more relevant than liver volume is liver fat reduction, because liver fat accumulation appears to be associated with cardiometabolic risk factors independent of obesity [31]. Magkos et al. could demonstrate in their randomized trial that weight loss disproportionately reduces liver fat. In that study, 5% weight loss reduces intrahepatic triglyceride by 13%; 11% weight loss reduced it by 52% and 16% by 65% [32]. In our study, liver fat after diet content dropped, determined by MRI, significantly of 18.35% over all patients; after diet with diet 2, the decline was higher with 21.51% vs. 14% after diet 1. Interestingly, the bigger the initial fat content is, the significantly bigger is the effect in terms of liver volume reduction. A comparable result was achieved by Haufe et al. [33]: patients with high baseline intrahepatic lipids (> 5.56%) lost ≈sevenfold more intrahepatic lipids compared with those with low baseline values (< 5.56%) after hypocaloric diet. Also, the results of Browning et al. support the assumption that very low-calorie diets have a particular impact on resolving hepatic lipid accumulation even after short-term diet [34, 35].

Interestingly, we could detect an increase of ALAT, ASAT and bilirubin opposite to a decrease of γGT and AP after 2-week VLCD. In addition, the greater the increase of ALAT or ASAT was, the greater was the liver volume reduction. Scragg et al. [36] observed in the first weeks of their 8-week VLCD the same effect of liver enzyme levels before the transaminases returned to baseline by week 4 and fell thereafter. The decrease of γGT was significantly in the first week before reaching a plateau after week 5. The cause of this acute increase in transaminases is not determined. Scragg et al. explain the increase in liver enzymes as consequence of an apoptosis of hepatocytes due to ingestion of free fatty acids which were caused by sudden massive lipolysis in adipose tissue. Following this explanation, the observed correlation between increase of transaminases and loss of liver volume would be conclusive. Also, our finding that a greater decrease of body fat was accompanied by a greater reduction of liver volume is conclusive following this assumption. But the hypothesis requires further investigation.

We collected in our study from almost all patients (*n* = 65) liver biopsies during surgery. The aim of this invasive procedure, although simple to implement surgically and without risk of complications (no post-bleeding was recorded), was to assess the extent of pathological liver changes in obese patients. We could confirm in nearly all patients with obesity NAFLD (98.5%), a transformation into NASH affected 53.8% of the patients. Fibrosis was detected in 95.4% of the biopsied patients, where fibrosis grade 1c (with (peri)portal fibrosis) was most common. In one patient, a prior unknown liver cirrhosis was identified. A liver biopsy prior to VLCD could not be performed for ethical concerns. In previous studies, a weight reduction of ≥ 7% was found to be histopathologically relevant [37]. We could confirm that the prevalence of NAFLD in patients with obesity is over 95% [38, 39], but more surprising was the finding that also in around 95% of our patients, fibrosis, even if mild, was present. The correlation between liver fat content measured by MRI and histopathology supports the used techniques in our study.

Remarkable is also the effect of the two hypocaloric diets on glucose and lipid metabolism. Triglycerides and LDL cholesterol dropped significantly after both diets and correlate with liver fat loss. Also, fasting blood glucose dropped (*p* = 0.028) and HbA1c deceased from 5.51 to 5.35, *p* < 0.001. Furthermore, regarding laboratory values, a significant decrease in inflammation is noticeable. This interesting effect is so far not investigated but should be further promoted. Over all data, significant differences between the two study groups were not detectable.

The compatibility of VLCD was good which led to a good compliance of the intake (daily documentation in the food diary). Forty-two per cent of the patients complained about mild to moderate side effects, the most common were obstipation, diarrhoea or abdominal pain. There was no discontinuation of the diet because of undesirable effects. In one case, the diet had to be switched from salty to sweet soups and shakes because of a chronic renal deficiency. Our results support findings of Colles et al. [16] and demonstrate a good tolerance of VLCDs.

One important limitation of our study is the lack of a control group. Therefore, effects of weight loss, circumferences drop or reduced liver volume on operation time, intraoperative blood loss or perioperative complications could not be compared to free-running preoperative conditions. However, we verified that both VLCDs are feasible and equally effective regarding weight loss, liver volume/liver fat reduction and improvements in parameters of inflammation, glucose and lipid metabolism. The detected decrease in inflammation and possible perioperative effects opens here new scientific approaches. Furthermore, the impact of low-energy diets prior to elective, non-bariatric surgery in patients with obesity is subject of ongoing research [40].
